# Development of subdomains in the medial pallium of *Xenopus laevis* and *Trachemys scripta*: Insights into the anamniote-amniote transition

**DOI:** 10.3389/fnana.2022.1039081

**Published:** 2022-11-03

**Authors:** Sara Jiménez, Nerea Moreno

**Affiliations:** Department of Cell Biology, Faculty of Biology, Complutense University of Madrid, Madrid, Spain

**Keywords:** hippocampus, medial cortex, telencephalon, evolution, evo-devo, amphibians, reptiles

## Abstract

In all vertebrates, the most dorsal region of the telencephalon gives rise to the pallium, which in turn, is formed by at least four evolutionarily conserved histogenetic domains. Particularly in mammals, the medial pallium generates the hippocampal formation. Although this region is structurally different among amniotes, its functions, attributed to spatial memory and social behavior, as well as the specification of the histogenetic domain, appears to be conserved. Thus, the aim of the present study was to analyze this region by comparative analysis of the expression patterns of conserved markers in two vertebrate models: one anamniote, the amphibian *Xenopus laevis*; and the other amniote, the turtle *Trachemys scripta elegans*, during development and in adulthood. Our results show that, the histogenetic specification of both models is comparable, despite significant cytoarchitectonic differences, in particular the layered cortical arrangement present in the turtle, not found in anurans. Two subdivisions were observed in the medial pallium of these species: a Prox1 + and another Er81/Lmo4 +, comparable to the dentate gyrus and the mammalian cornu ammonis region, respectively. The expression pattern of additional markers supports this subdivision, which together with its functional involvement in spatial memory tasks, provides evidence supporting the existence of a basic program in the specification and functionality of the medial pallium at the base of tetrapods. These results further suggest that the anatomical differences found in different vertebrates may be due to divergences and adaptations during evolution.

## Introduction

Although there is currently an intense debate about the subdivisions that constitute the pallial region, their derivatives and boundaries (Abellán et al., 2014; Puelles, 2017, 2021; Desfilis et al., 2018; Medina et al., 2021), numerous studies have demonstrated similarities between the mammalian hippocampal formation (HF) and derivatives of the medial pallium (MP) in the rest of vertebrates [for review see Butler (2017)] speaking for the homology of these structures. Topologically, the mammalian HF is located in the mediodorsal area of the telencephalon, bordered rostrally by the indusium griseum and the dorsal tenia tecta (Wyss and Sripanidkulchai, 1983; Künzle, 2004; Laplante et al., 2013). In adults, this cortical structure is composed of a central region, that shows a characteristic C-shape organized in three layers and the parahippocampal region [for a review see Amaral and Lavenex (2009)]. Similarities at the functional, hodological and cellular levels have been proposed between the HF and the MP of the rest of the amniotes (Filimonoff, 1964; Karten and Hodos, 1967; Bingman and Yates, 1992; Striedter, 1997; Puelles et al., 2000; Lohmann et al., 2004; Atoji and Wild, 2006; Kahn and Bingman, 2009; Mayer et al., 2013; Abellán et al., 2014; Naumann et al., 2015; Reiter et al., 2017; Gupta et al., 2020; Schede et al., 2021). But it is mostly in mammals where the neurogenesis of this region has been extensively studied (Sugiyama et al., 2013; Zhang and Jiao, 2015) and specifically the maintenance of this neurogenic capacity in adults (Alvarez-Buylla and Lim, 2004; Gould, 2007; Kempermann et al., 2015). In anamniotes this territory has also been analyzed. Specifically, in fish and amphibians, demonstrating the functional involvement of the MP in learning and navigation strategies (Salas et al., 2006; Sotelo et al., 2016; Bingman and Muzio, 2017; Liu et al., 2020; Rodríguez et al., 2021). In amphibians, the organization of the MP is known to be simple, without layers or nuclei, although the existence of subdivisions has been proposed based on their connectivity (Neary, 1990; Northcutt and Ronan, 1992; Roth and Westhoff, 1999; Westhoff and Roth, 2002; Roth et al., 2007). In addition, more recent genoarchitectural analyses in amphibians have shown that the MP expresses conserved transcription factors involved in mammalian hippocampal development (Moreno et al., 2004; Lust et al., 2022; Woych et al., 2022).

In this context, although evolutionary knowledge of this region has increased significantly in recent years (Abellán et al., 2014; Medina et al., 2017; Tosches et al., 2018; Woych et al., 2022), we still lack a detailed comparative analysis of the expression pattern of conserved MP developmental genes (usually used as markers to identify the mammalian MP) during development and in the adult in non-mammalian species.

Thus, it is highly desirable to add missing information on developmental and adult brain genoarchitectonics in the already well-studied *Xenopus laevis* to fill up gaps for the generally underinvestigated amphibians. Furthermore, we aimed to have a direct comparison to amniotes and chose the turtle, *Trachemys scripta elegans*, the most abundant of three subspecies of this new world turtle species. In addition to available data in lizards (Desfilis et al., 2018), there are cell type data analyzed by RNAseq (Tosches et al., 2018). Therefore, the main objective of the present study was to comparatively analyze the development and adult organization of this region, by analyzing its cytoarchitecture and studying the expression pattern of medial pallial markers in both selected models. Our results allow us to establish the relationship between different areas of the MP of anurans and amniotes.

## Materials and methods

### Animals and tissue processing

Adults *Xenopus laevis* (anuran amphibian) were purchased from the European Xenopus Resource Centre (EXRC; EXRC@xenopusresource.org). The embryonic and larvae stages [from (Faber and Nieuwkoop, 1956)] were obtained from *in vitro* fertilizations in the laboratory. Developing [st 17–26 from (Greenbaum, 2002)] and young adults of *Trachemys scripta elegans* were obtained in collaboration with the Centro de Conservación de Especies Dulceacuícolas de la Comunidad Valenciana (CCEDCV).

Adult and developing *Xenopus* were anesthetized by immersion with 0.1% tricaine methanesulfonate solution (MS222, pH 7.4; Sigma-Aldrich, Steinheim, Germany). The turtles were anesthetized by intraperitoneal injections with sodium pentobarbital (50–100 mg/kg, Normon Labs, Madrid, Spain). Developing stages were fixed by immersion, and adults by transcardial perfusion with the fixative solutions: 4% paraformaldehyde in a 0.1 M phosphate buffer (PB, pH 7.4) for the immunohistochemical techniques and MEMFA (0.1 M MOPS [4 morpholinopropanesulphonic acid], 2 mM ethylene glycol tetraacetic acid, 1 mM MgSO4, 3.7% formaldehyde) for the *in situ* hybridization. The brains were subsequently cryoprotected in a solution of 30% sucrose in PB for 4–6 h at 4°C, and cut at 30–40 μm thickness, in transverse or sagittal planes.

### Immunofluorescence and *in situ* hybridization analysis and imaging

Single and combined immunohistochemical reactions were performed on free-floating sections, using the primary antibodies, diluted in PB containing 0.5% Triton X-100, described in the Table 1 (see Table 1 for commercial specifications, immunogen information, and used dilution). The second incubations were conducted with the appropriately labeled secondary antibody (see specifications in the Table 1 figure legend). Antibodies controls included omission and incubation with preimmune mouse or rabbit sera instead of the primary antibody. No residual staining was observed in any case.

**TABLE 1:** List of primary and secondary antibodies used, immunogen, commercial supplier and dilution.

Name	Immunogen	Commercial supplier	Dilution
BLBP	Synthetic peptide conjugated to KLH derived from within residues 1–100 of mouse BLBP	Polyclonal rabbit anti-BLBP. Abcam. Catalog reference: ab32423	1:500
Ctip2	150 amino acids located at the extreme amino terminus of the protein, CTIP2 (1–150)	Monoclonal rabbit anti-Ctip2. Absolute antibody. Catalog reference: Ab00616-23.0	1:500
Cux2	Synthetic peptide within Human Cux2 aa 1,000-1,100 conjugated to keyhole limpet haemocyanin	Polyclonal rabbit anti-Cux2.Abcam. Catalog reference: ab216588	1:100
DCX	Epitope mapping at the C-terminus of Doublecortin of human origin	Polyclonal goat anti-DCX. Santa Cruz. Catalog reference: sc-8066	1:500
GFAP	GFAP isolated from cow spinal cord	Polyclonal rabbit anti-GFAP. Invitrogen. Catalog reference: PA5-16291	1:100
Lhx2	Amino acids: 231–406 of human LIM/homeobox protein Lhx2	Monoclonal mouse anti-Lhx2. Developmental Studies Hybridoma Bank. Catalog reference: 2C10	1:100
Lmo4	Full length human LMO4 recombinant protein (UniProt: P61968)	Polyclonal rabbit anti-LMO4. GeneTex. Catalog reference: GTX129616	1:100
Pax6	Peptide sequence: QVPGSEPDMSQYWPRLQ of the C-terminus of mouse PAX6 protein	Polyclonal rabbit anti-PAX6. Covance. Catalog reference: PBR-278	1:250
PH3	Amino acid sequence containing phosphorylated Ser 10 of Histone H3	Polyclonal rabbit anti- p-Histone H3 (Ser 10)-R. Santa Cruz. Catalog reference: sc-8656-R	1:500
Prox1	Synthetic peptide from the C-terminus of mouse Prox1	Polyclonal rabbit anti-Prox1. Millipore. Catalog reference: AB5475.	1:1,000
Satb1/2	Aminoacid from 601 aa at the C-terminus of isoform 1 human SATB2 protein	Mouse monoclonal anti-Satb2. Abcam. Catalog reference: ab51502	1:100
Sox2	Synthetic peptide conjugated to KLH derived from within residues 300 to the C-terminus of Human SOX2.	Polyclonal rabbit anti-Sox2. Abcam. Catalog reference: ab97959	1:500
Tbr1	Amino acids 1-200 at the N-terminus of mouse TBR-1	Polyclonal rabbit anti-Tbr-1. Santa Cruz Biotechnology. Catalog reference: sc-48816	1:500

Secondary antibodies used; Alexa 594-conjugated goat anti-rabbit (red fluorescence; Invitrogen; catalog reference #A-11012), Alexa 488-conjugated goat anti-mouse (green fluorescence; Invitrogen; catalog reference #A-11001), Alexa 594-conjugated chicken anti-goat (red fluorescence; Invitrogen; catalog reference #A-21468), Alexa 488-conjugated chicken anti-rabbit (green fluorescence; Invitrogen; catalog reference #A-21441).

Plasmids were linearized with the appropriate restriction enzymes (Promega, Madison, WI, USA), used as templates for RNA synthesis (T3, T7 or SP6 polymerase; Promega; see Table 2). Riboprobes were synthesized in presence of digoxigenin-11-UTP (Roche Diagnostics, Mannheim, Germany). The protocol for single chromogenic *in situ* hybridization was modified from (Ferran et al., 2015). Double *in situ* hybridization and immunofluorescence analyses were carried out in sequential steps, in which *in situ* hybridizations were first developed, photographed and subsequently immunofluorescence was performed.

**TABLE 2:** List of gene markers used, sequence of the gene in the construct, origin of the plasmid and enzymes employed to synthesize the antisense riboprobe.

Gene	GenBank ref. sequence	Origin	Plasmid	Linearization enzyme and polymerase
xEGFR	XM_041566091.1	Dr. Chenbei Chang. Department of Cell Biology, The University of Alabama at Birmingham. Alabama, Birmingham.	pCS105	*Hpa*I*/T3*
xEr81	AF057565.1	Dr. Herbert Steinbeisser. Max-Planck-Institute for Developmental Biology. Tübingen, Germany.	pSPORT 1	*Sal*I/SP6
xMef2c	NM_001092414.1	Dr. Susanne J Kühl. Institute of Biochemistry and Molecular Biology, Ulm University, Ulm, Germany.	pSC-B	*Spe*I*/T3*
xLmo4	NM_001094421.2	EXRC Clone Number 776. IMAGE: 4032190	pCMV-Sport6	*Sal*I/T7
tEr81	XM_042856658.1	Dr. Laurent Guilles. Max-Planck-Institute for Brain Research. Frankfurt, Germany	PCR^®^ II	*Not*I/SP6

The sections were analyzed with the microscopes: Olympus BX51 microscope, Olympus FV 1200 and Leica sp-2 AOBS confocal. The figure preparation was done with Adobe Photoshop CS6 (Adobe Systems, San Jose, CA) and Canvas X (ACD Systems, Canada).

## Results

### The medial pallium of *Xenopus laevis* and *Trachemys scripta elegans*

The distribution pattern of the markers used in this study has been analyzed in the developing and adult pallium of two tetrapod models: the anamniote amphibian *Xenopus laevis* and the amniote turtle *Trachemys scripta elegans*. The nomenclature used in this study is based on previous neuroanatomical studies (Desan, 1984; Neary, 1990; Ulinski, 1990; Roth and Westhoff, 1999; Moreno et al., 2004, 2008; Moreno and González, 2017; Puelles, 2017; Tosches et al., 2018). The RNA probes and antibodies used (see Tables 1, 2) revealed distinct and consistent patterns from animal to animal and comparable between the different stages of the two species used.

**FIGURE 1:**
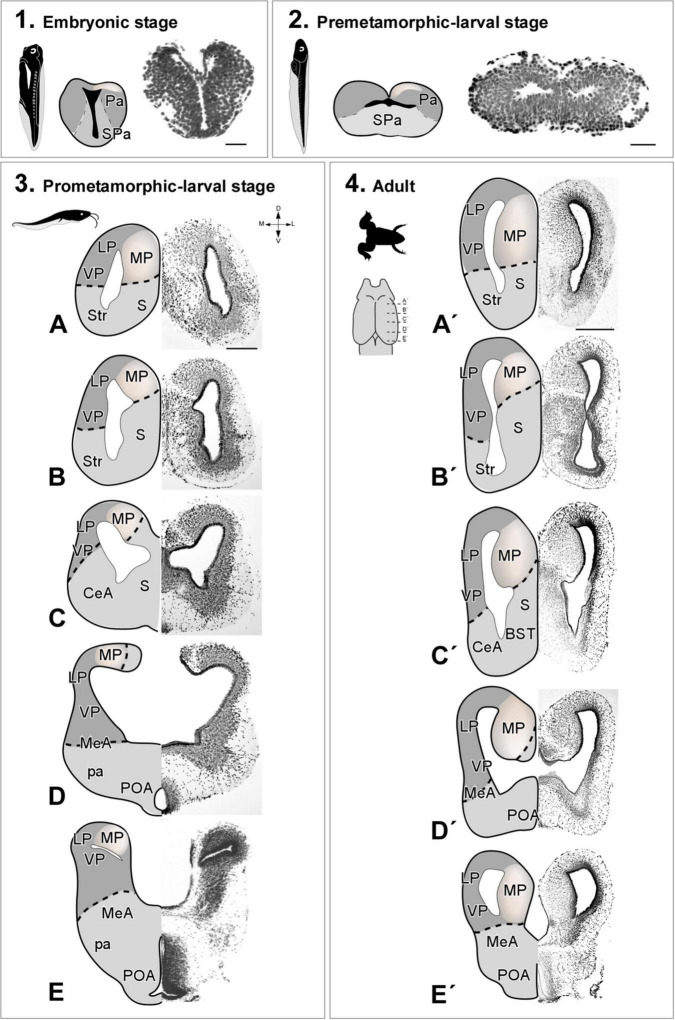
Photomicrographs of transverse sections through the telencephalon of *Xenopus laevis* at embryonic **(1)**, premetamorphic **(2)**, prometamorphic larvae stages **(3A–E)** and adult **(4A′–E′)**. Nissl staining allowed the anatomical identification of the medial pallium during embryonic stages prior to telencephalic evagination **(1)** or after evagination in premetamorphic **(2)**, prometamorphic **(3)** larval stages and adult **(4)**. The region of the medial pallium is pink-colored in the schematic drawings of telencephalon semi-hemispheres. Scale bar in **1**, **2**, **(3A–E)** = 200 μm, **(4A′–E′)** = 500 μm. See the abbreviation list.

**FIGURE 2:**
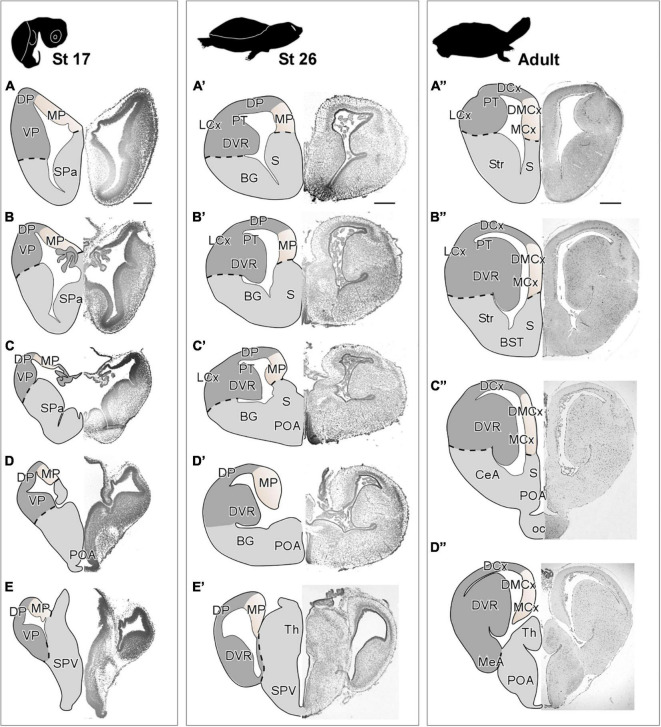
Photomicrographs of transverse sections through the telencephalon of *Trachemys scripta elegans* at st 17 **(A–E)**, st 26 **(A′–E′)** and adult **(A”–D”)**. Nissl staining allowed the anatomical identification of the medial pallium during early **(A–E)** or late **(A′–E′)** embryonic stages and adult **(A”–D”)**. The region of the medial pallium is pink-colored in the drawings of telencephalon semi-hemispheres. Scale bar = 500 μm. See the abbreviation list.

The cellular organization of the pallium of *Xenopus laevis* (Figure 1) and *Trachemys scripta elegans* (Figure 2) was visualized in coronal rostro-caudal telencephalic sections of both models by basic Nissl cytological staining. The telencephalon of *X. laevis* dramatically changes from embryonic to premetamorphic larval stages (Figure 1), suffering at this developmental time the major evagination events, and the determinant waves of neurogenesis [see Moreno and González (2017) for further details]. From the prometamorphic larval stages (Figures 1A–E) the MP was distinguishable, showing an aspect similar to that found in the adult (Figures 1A′–E′), but with a more dorsalized anatomical location, due to the pallial expansion process of this developmental stage.

In *T. scripta*, the MP constitutes the most medial portion of the pallium, showing a three-layer arrangement, with a main cell layer, surrounded by two layers of very low cell density. At embryonic stages (stage 17), the MP showed a highly developed ventricular zone and a discrete number of cells in the mantle (Figures 2A–E). Later in development, at stage 26 just before hatching (Figures 2A′–E′), the anatomy of the pallium closely resembles that found in the adult, and a layered organization can be observed in the region adjacent to the ventricle. At this moment, the major cellular layer observed is close to the ventricular surface, whereas adjacent to it, at the mantle layer, the cellular density is very low. At this later the development stage, in the medial cortex (MCx) the cell density increases in the mantle, while the ventricular zone becomes thinner, due to the expansion in this area and, in the adjacent dorsal pallium, apparently, the number of mantle cells also increases. In the adult, MCx and dorsomedial cortex (DMCx) cells are organized mainly in a main layer near the ventricle (Figures 2A′′–D′′). The number of cells in the mantle is sparse but more numerous in the case of DMCx.

### The radial glia cells in the medial pallium of *Xenopus laevis* and *Trachemys scripta elegans*

**FIGURE 3:**
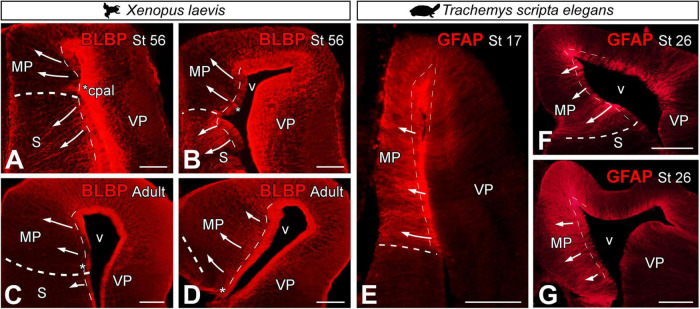
Photomicrographs of transverse sections through the telencephalon of *Xenopus laevis* **(A–D)** and *Trachemys scripta elegans* **(E–G)** at the stages indicated in each photomicrograph, showing the direction of the radial glia in both models (arrows) by BLBP in *X. laevis* and GFAP in *T. scripta*. The asterisks indicate the pallial commissure. The thin dotted lines point to the ventricular surface and the thick dotted lines to the pallium-subpallium boundary. Scale bar = 200 μm. See the abbreviation list.

Analysis of radial glial cells in the MP was performed using two markers: brain lipid binding protein (BLBP) in *Xenopus laevis* (Figures 3A–D) and glial fibrillary acidic protein GFAP in *Trachemys scripta elegans* (Figures 3E–G). Both markers are widely described in the literature as markers of radial glia, and each one worked most satisfactorily in these species, although these markers may not initiate their expression simultaneously in all regions of the telencephalon [see Docampo-Seara et al. (2019)]. In X. *laevis*, the direction of radial glia fibers during pallial development (Figures 3A,B) and in the adult (Figures 3C,D) was perpendicular to the ventricular lining. Coronal sections of the telencephalon of *X. laevis* prometamorphic larvae showed that the direction of the radial glia fibers in the MP curves slightly dorsally, reaching the pial surface, in the opposing direction to that observed in the subpallial region (see arrows in Figures 3A,B).

In *T. scripta*, the direction of the radial glia fibers in both developmental stages analyzed and in the adult was perpendicular to the ventricular lining, connecting it with the pial surface (Figures 3E–G).

### Lhx2 as a progenitor cell marker in the medial pallium of *Xenopus laevis* and *Trachemys scripta elegans*

It is widely described in the literature that the LIM-homeodomain transcription factor Lhx2 is expressed in mouse (Bulchand et al., 2003; Roy et al., 2014), chicken (Abellán et al., 2014), lizard (Desfilis et al., 2018), and amphibian (Moreno and González, 2017) MP progenitors and in postmitotic cells in the adult.

**FIGURE 4:**
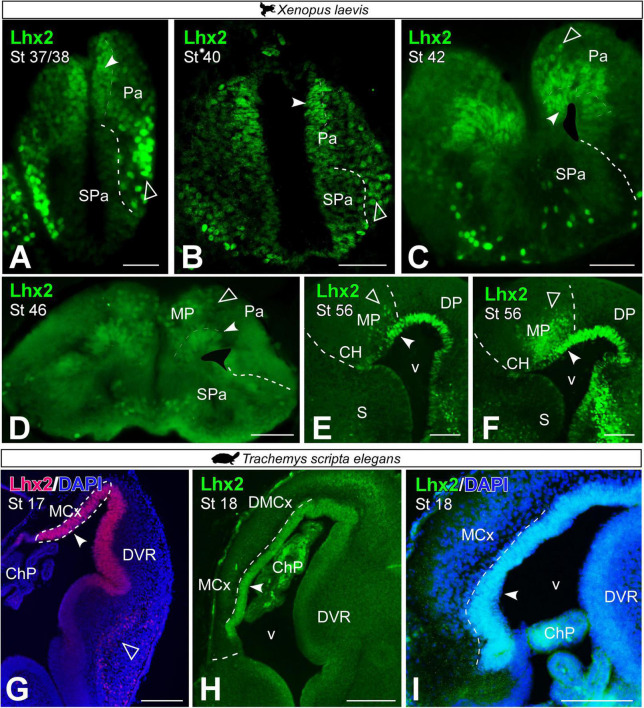
Photomicrographs of transverse sections through the telencephalon of *Xenopus laevis* **(A–F)** and *Trachemys scripta elegans* **(G–I)** of Lhx2 expression in the medial pallium at embryonic **(A–C,G–I)** and early **(D)** and late larvae stages **(E,F)**. In each panel the developmental stage is indicated. Filled arrowheads point to ventricle expressing cells and empty arrowheads to mantle expressing cells. Scale bar **(A–H)** = 200 μm and **(I)** = 100 μm. See the abbreviation list.

From embryonic stages, before telencephalic evagination (Figures 4A,B), Lhx2 expression was observed in the ventricular region of the *X. laevis* pallium (see filled arrowhead in Figures 4A,B) and in postmitotic cells in the mantle, in ventrolateral position, which showed increased intensity in labeling (see empty arrowhead in Figures 4A,B). This ventricular expression was maintained throughout the embryonic up to larval stages (Figures 4C–F), at different stages of the telencephalic evagination process. From late embryonic stages (st 42; Figure 4C), when the evagination process begins, Lhx2 + cells separated from the ventricle began to be observed in the medial-dorsal region of the pallium (see empty arrowheads in Figures 4C–F). From larval stages, this population of postmitotic cells in the MP increased markedly (Figure 4D), occupying a large territory in the mid-dorsal telencephalic region. In late larval stages, it was further observed that these progenitor cells followed a marked rostrocaudal pattern, being more abundant in the caudal telencephalic regions (Figures 4E,F).

In *T. scripta*, Lhx2 + ventricular cells were observed in the MP in the embryonic stages analyzed (Figures 4G–I). In these stages, no Lhx2 + cells were observed in the mantle outside the ventricular region of the MP.

### Analysis of progenitors in the medial pallium of *Xenopus laevis* and *Trachemys scripta elegans*

The analysis of progenitor markers allowed us to study the development of the MP from early stages, st 46 in *Xenopus laevis* and st 17 in *Trachemys scripta elegans*, when the process of pallial evagination has already begun.

**FIGURE 5:**
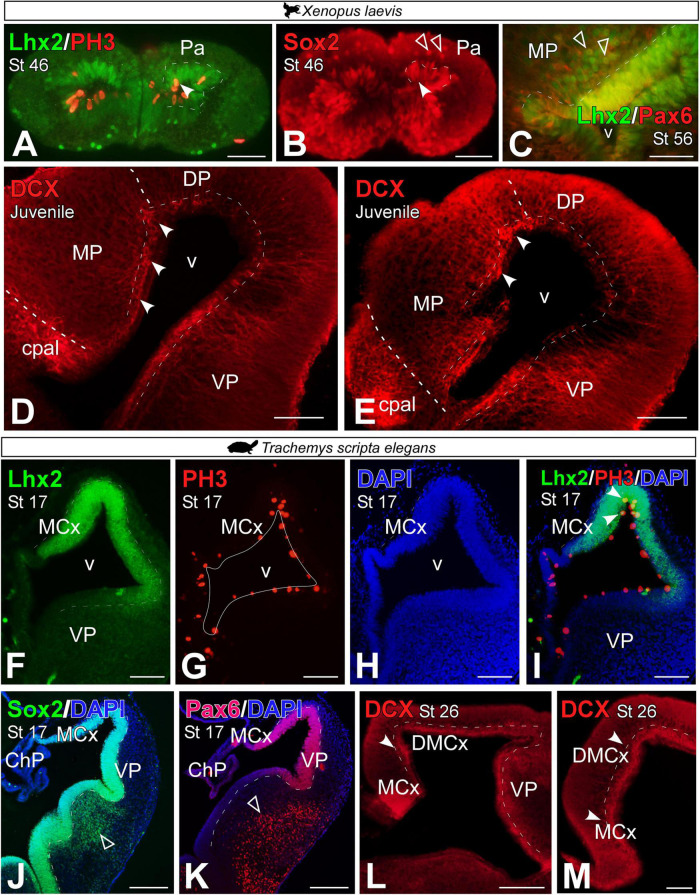
Photomicrographs of transverse sections through the developing telencephalon of *Xenopus laevis* **(A–E)** and *Trachemys scripta elegans* **(F–M)** showing the distribution of Lhx2 in combination with PH3, where only double-labeled mitotic cells are observed in the ventricular zone (arrowheads). The progenitor cell marker Sox2 **(B,J)** is observed in both cases in the ventricular region (filled arrowhead) and in *X. laevis* at these stages additionally in the mantle (empty arrowhead). The Lhx2 cells are proximal to the ventricle co-expressed Pax6 in the ventricle (filled arrowhead) and out of it [empty arrowheads in panel **(C)**]. The neuroblast marker doublecourtine **(D,E,L,M)** was detected in the ventricular region of the medial pallium in both cases (filled arrowheads). In each panel, the developmental stage and the color code for the used markers are indicated. Filled arrowheads point to ventricle-expressing cells and empty arrowheads to mantle-expressing cells. Scale bar in panels **(A,B,D–L)** = 200 μm and **(C,M)** = 100 μm. See the abbreviation list.

In *X. laevis*, in premetamorphic larvae, we observed that some of the ventricular Lhx2 + cells expressed the mitotic marker Phospho-Histone H3 (PH3; see arrowhead in Figure 5A). As for the expression of Sox2 in the MP, this neuroblast marker was found in the ventricular region of the MP and in scattered cells in the subventricular zone of this domain, from larval stages (see filled and empty arrowheads respectively in Figure 5B). From prometamorphic stages of *X. laevis*, in addition to ventricular expression, Lhx2 cells proximal to the ventricle co-expressed Pax6, mostly in the ventral domain (see empty arrowheads in Figure 5C). Doublecortin (DCX), a neuroblast marker described in the *X. laevis* pallium [see Moreno and González (2017)], was identified in the MP of juvenile individuals, and with particular intensity in the dorsal region of this portion at the medial and caudal level of the telencephalon (see arrowheads in Figures 5D,E).

In *T. scripta*, during development, some Lhx2 ventricular cells in the MP region were PH3 + (Figures 5F–I, see arrowhead in Figure 5I). At st 17, virtually all ventricular cells of the pallium, and particularly the MP, expressed Sox2 (Figure 5J) and Pax6 (Figure 5K). However, in these stages, no subventricular cells for any of these markers were found outside the ventricle, unlike in the subpallial region (see empty arrowheads in Figures 5J,K). The expression of DCX was detectable from late developmental stages, st 26, in the medial and dorsomedial cortex of the turtle (see arrowheads in Figures 5L,M).

### Prox1 expression in the medial pallium

**FIGURE 6:**
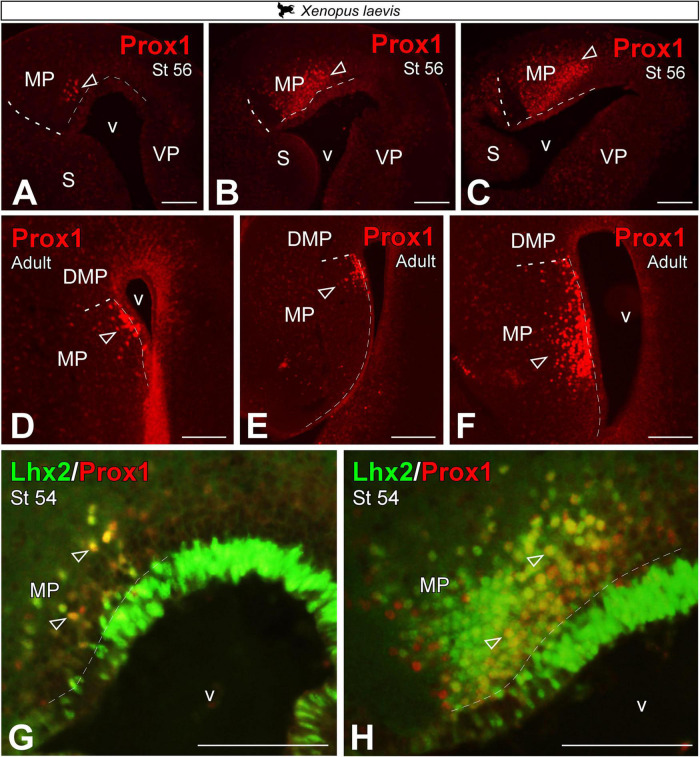
Photomicrographs of transverse sections through the telencephalon of *Xenopus laevis* at larval stages **(A–C)** and adult **(D–F)** showing, from rostral to caudal levels, Prox1 expression **(A–F)** and its combination with Lhx2 **(G,H)**. In each panel, the developmental stage and the color code for the used markers are indicated. Empty arrowheads point to mantle-expressing cells. Scale bar = 200 μm. See the abbreviation list.

It has been described in the literature that the gene Prospero homeobox (*Prox1*) defines the dentate gyrus of the HF in mammals [DG; (Jessberger et al., 2008)]. During the early development of *X. laevis*, in embryonic and premetamorphic larval stages, no Prox1 + cells were detected (data not shown). From prometamorphic larval stages, st 54, Prox1 expression was observed in a subregion of the MP, with a clear rostro-caudal increasing pattern of cell density (Figures 6A–C). The same pattern of expression was observed in the adult, where the expression was restricted to the most ventral portion of the MP, which is narrower at rostral levels but expands to occupy almost the entire length of the MP at caudal levels (Figures 6D–F). In addition, we observed that most Prox1 + cells co-localize with Lhx2 + cells adjacent to the ventricle of the MP (see empty arrowheads in Figures 6G,H).

**FIGURE 7:**
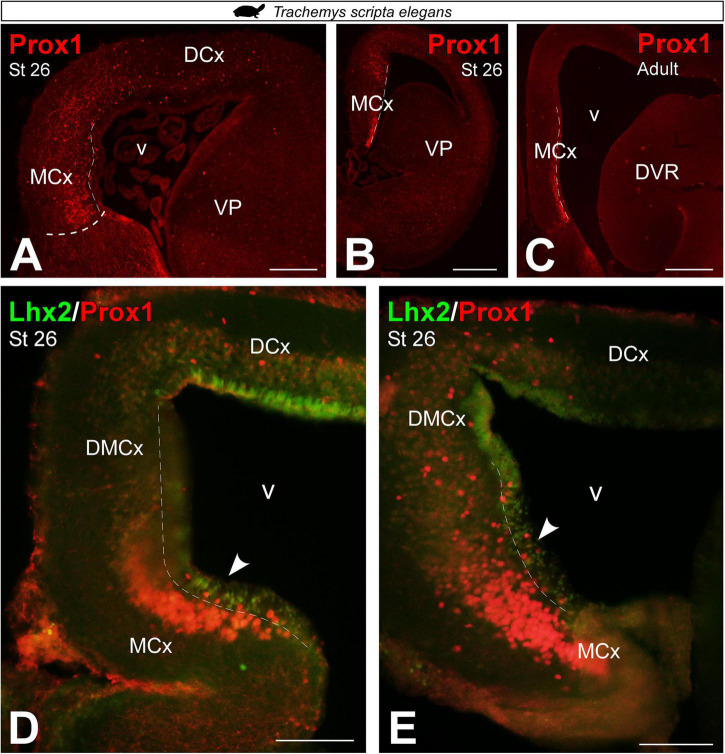
Photomicrographs of transverse sections through the telencephalon of *Trachemys scripta elegans* showing, from rostral to caudal levels, Prox1 expression **(A–C)** and its combination with Lhx2 **(D,E)**. In each panel, the developmental stage and the color code for the used markers are indicated. Filled arrowheads point to ventricle-expressing cells. Scale bar in panels **(A,C,D,E)** = 200 μm and **(B)** = 500 μm. See the abbreviation list.

In *T. scripta*, Prox1 telencephalic expression was also not detected at very early developmental stages, st 17 (data not shown). Whereas in st 26, the expression of Prox1 in the MP domain was especially concentrated in its ventral part, the MCx, where it occupied a large territory in the mantle (Figures 7A,B). Similarly, in the adult we found Prox1 expression restricted to its ventral part, the MCx (Figure 7C). Finally, at late developmental stages, st 26, expression of Prox1 in combination with Lhx2 did not show subventricular cells expressing both markers, as at this stage Lhx2 expression was reduced to the ventricle (see arrowheads in Figures 7D,E).

### Er81 and Lmo4 expressions in the medial pallium

**FIGURE 8:**
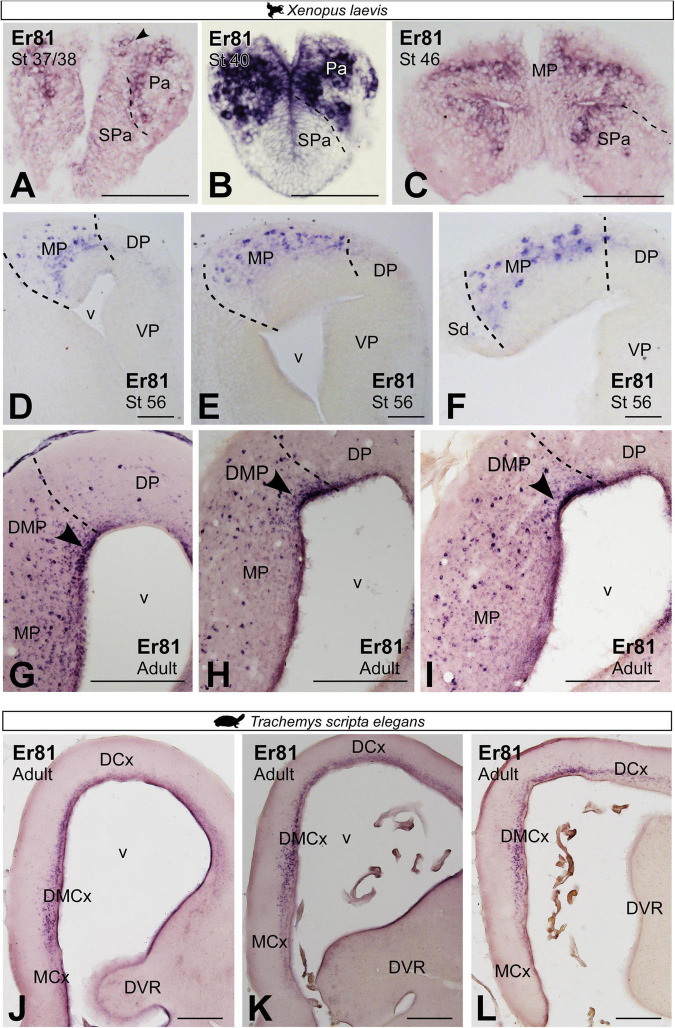
Photomicrographs of transverse sections through the telencephalon of *Xenopus laevis* **(A–I)** and *Trachemys scripta elegans* **(J–L)** showing, from rostral to caudal levels, the Er81 expression at embryonic **(A)**, premetamorphic **(B,C)** and prometamorphic larval *X. laevis* stages **(D–F)** and adults **(G–I)** and adults *Trachemys scripta elegans* **(J–L)**. Filled arrowheads point to ventricle expressing cells. Scale bar in panels **(A–F,J–L)** = 200 μm and **(G–I)** = 500 μm. See the abbreviation list.

In mammals, ETV1 (Er81) transcription factor and LIM domain only 4 (Lmo4) are expressed in the mammalian HP (Lakhina et al., 2013; Bienkowski et al., 2018). In *X. laevis*, Er81 expression from embryonic stages, st 37/38 and st 40, to premetamorphic larval stages, st 46, was localized throughout the telencephalon including the pallial region (Figures 8A–C). From prometamorphic larval stages, st 54/56, the expression of Er81 was mostly restricted to the MP, occupying this entire pallial domain from rostral to caudal levels (Figures 8D–F). In adults, the Er81 expression pattern allowed the identification of at least two medial pallial subdivisions. A dorsal portion, where the ventricular expression of Er81 was more intense, denominated dorsomedial pallium (DMP; see arrowheads in Figures 8G–I). Meanwhile, in the rest of the domain, the proper MP, numerous Er81 + cells were located scattered throughout the mantle (Figures 8G–I). In adult *T. scripta*, Er81 expression in the MP region was restricted to the most dorsal domain called the dorsomedial cortex (DMCx; Figures 8J–L).

**FIGURE 9:**
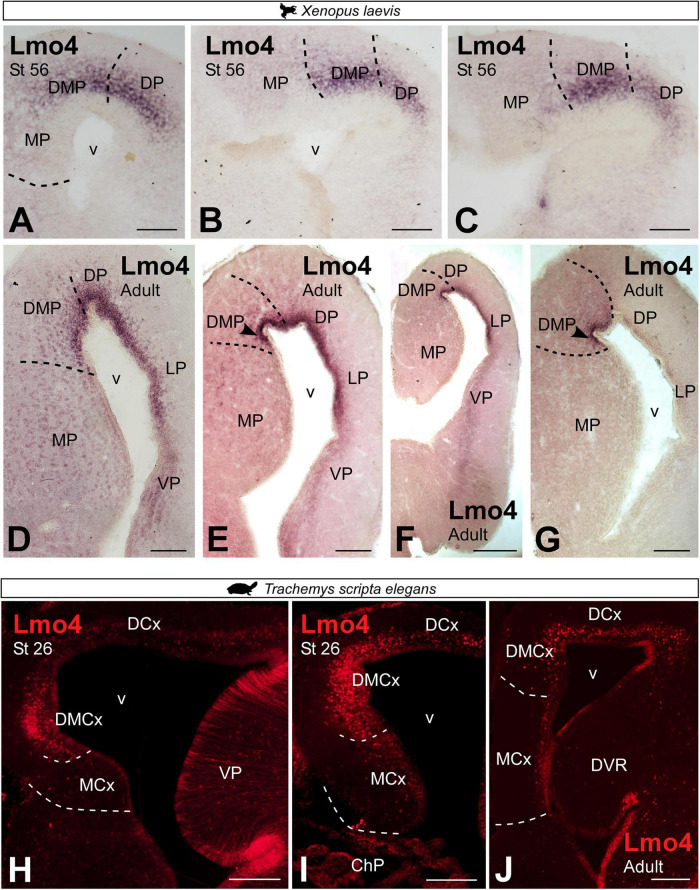
Photomicrographs of transverse sections through the telencephalon of *Xenopus laevis* **(A–G)** and *Trachemys scripta elegans* **(H–J)** showing, from rostral to caudal levels, the Lmo4 expression at developmental stages **(A–C,H, I)** and adults **(D–G,J)**. Scale bar in panels **(A–E,G–J)** = 200 μm and **(F)** = 500 μm. See the abbreviation list.

In *X. laevis*, Lmo4 expression in prometamorphic larval stages was localized rostrocaudally in the most dorsal region of the MP, formerly referred to as the DMP, as well as in the adjacent pallial region, the dorsal pallium (Figures 9A–C). The pattern of Lmo4 expression in the adult was maintained, showing continued rostrocaudal expression, in the dorsal portion of the MP, the DMP (Figures 9D–G). In adult *T. scripta*, Lmo4 expression was observed in the most dorsal domain of the medial pallium, the DMCx, in addition to the adjacent dorsal cortex (Figures 9H–J). In both regions, a dense layer of cells was located close to the ventricle.

**FIGURE 10:**
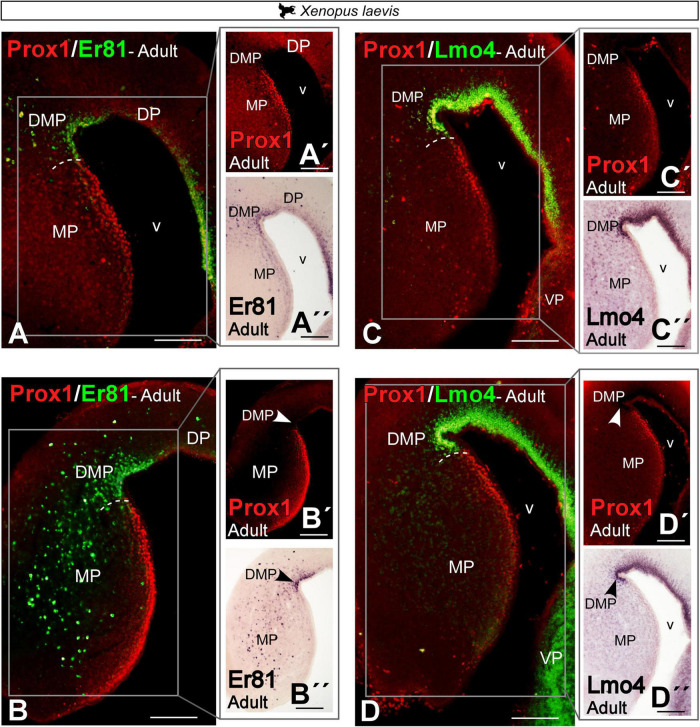
Photomicrographs of transverse sections through the telencephalon of *Xenopus laevis* (A–D) showing, from rostral to caudal levels, the Prox1/Er81 (A,B) and the Prox1/Lmo4 expressions (C,D). The white box in the figures (A–D) corresponds to the magnifications shown in the adjacent photos with the single staining (A′–D′,A′′–D′′). In each panel, the color code for the used markers is indicated. Scale bar = 200 μm. See the abbreviation list.

Double labeling in *Xenopus laevis* of Prox1 with Er81 and with Lmo4 (Figures 10A–D,A′–D′,A′′–D′′) confirmed the previously described expression pattern (see Figures 6, 8, 9) and the clear identification of the boundary between MP and DMP.

### Additional markers: Ctip2, Cux2, Mef2c EGFR, and Satb1/2

We have analyzed additional markers described in the HF of amniotes. This is the case of Ctip2, Cux2, Mef2c, EGFR and Satb1/2.

**FIGURE 11:**
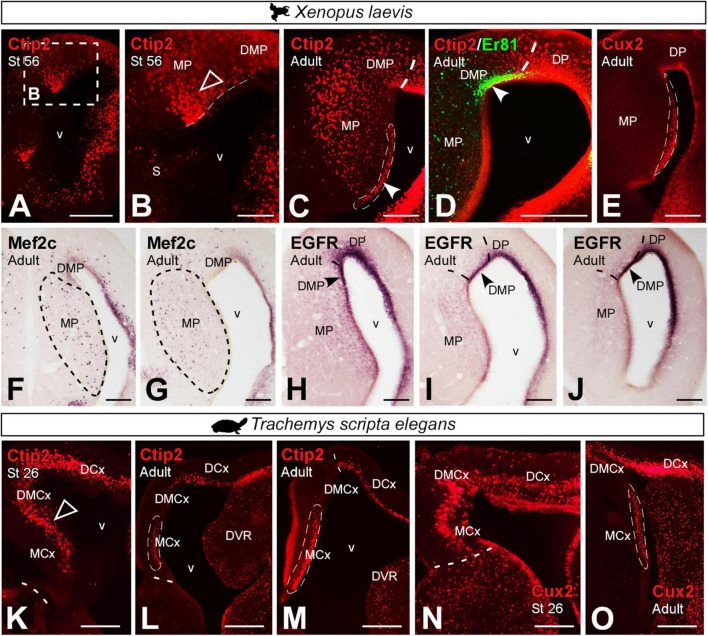
Photomicrographs of transverse sections through the developing and adult telencephalon of *Xenopus laevis* **(A–J)** and *Trachemys scripta elegans* **(K–O)**. Ventricular expression is detected in the MP (filled arrowheads) and mantle (empty arrowheads), in prometamorphic larvae **(A,B)** of *Xenopus* and, adult **(C)**. This localization is confirmed by the combination of Ctip2 with Er81, detected in the DMP **(D)**. Expression of Cux2 **(E)**, Mef2c **(F,G)** and EGFR **(H–J)** are localized in adult *X. laevis* in the MP region. Ctip2 expression in developing *T. scripta* is observed in MCx and in DMCx **(K)**, whereas in the adult was observed mainly in the MCx **(L,M)**, as well as Cux2 in developing **(N)** and in adults **(O)**. In each panel, the developmental stage and the color code for the used markers are indicated. Filled arrowheads point to ventricle-expressing cells and empty arrowheads to mantle-expressing cells. Scale bar in panels **(A,C–O)** = 200 μm and **(B)** = 100 μm. See the abbreviation list.

In prometamorphic *X. laevis* larvae, Ctip2 expression was detected throughout the MP, both in the ventricle and mantle (Figures 11A,B). In adult *X. laevis*, a significant population of Ctip2 + cells was observed in the ventricular zone of the MP (see arrowhead in Figure 11C), lacking in the ventricle of the DMP (Er81 +; see arrowhead in Figure 11D), as well as in the mantle of both regions. Cux2 expression in adult *X. laevis* was observed in the ventricular domain of the MP, avoiding the adjacent DMP (Figure 11E). Mef2c was expressed by scattered cells in the MP, absent in the DMP (Figures 11F,G). And ventricular EGFR expression was observed in the DMP, lacking in the MP (see arrowhead in Figures 11H–J). In *T. scripta*, Ctip2 (Figures 11K–M) and Cux2 (Figures 11N,O) expressions in development was extensive throughout the MP (Figures 11K,N), but in adults it was confined rostrocaudally to the ventricular region of medial cortex, being higher at caudal levels (Figures 11L,M,O).

**FIGURE 12:**
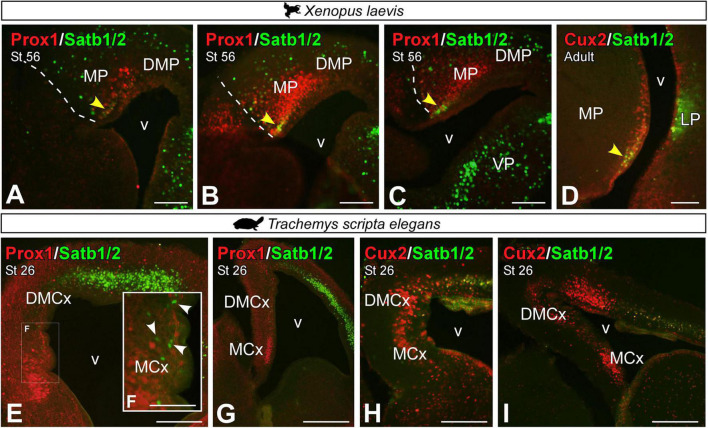
Photomicrographs of transverse sections through the developing and adult telencephalon of *Xenopus laevis* **(A–D)** and *Trachemys scripta elegans* **(E–I)** showing the combined expression of Satb1/2 with Prox1 **(A–C,E–G)** and Cux2 **(D,H,I)**. In each panel, the developmental stage and the color code for the used markers are indicated. Yellow arrowheads **(A–D)** indicate double-labeled cells. The white box in Figure **(E)** corresponds to the magnified photo in panel **(F)**. The white arrowheads point to Satb1/2 positive cells, no double-labeled. Scale bar in panels **(A–E,G–I)** = 200 μm and **(F)** = 100 μm. See the abbreviation list.

Combined expression of Prox1 and Satb1/2 in the MP of premetamorphic *X. laevis* larvae (Figures 12A–C) identified that at least one subpopulation of Prox1 cells in the most ventral portion of the MP coexpress Satb1/2 (see yellow arrowhead in Figures 12A–C). Similarly, in the adult, these Satb1/2 cells were found to coexpress Cux2 in the most ventral portion (see yellow arrowhead in Figure 12D). Whereas in the turtle, Satb1/2 was hardly expressed in the MP derivatives, except for a few scattered cells (Figures 12E–I), especially in DMCx (see arrowheads in Figure 12F), these cells also did not express Cux2 (Figures 12H,I), thus this subpopulation is absent in the medial pallial region of turtles.

## Discussion

Research on the evolution of the pallial region in vertebrates has proposed that ∼320 million years ago, the common living ancestor of amniotes already possessed a cortical region organized in three layers, a similar structure to the current mammalian hippocampus and/or the reptilian cortex (Aboitiz and Montiel, 2007; Aboitiz, 2011; Aboitiz and Zamorano, 2013; Roy et al., 2014; Naumann et al., 2015; Reiter et al., 2017). This goes further, as this common ancestor to amniotes may actually be closer to the current pallial organization of amphibians, at the base of the diversification between amniotes and anamniotes (amphibians constitute the only extant anamniote tetrapods). Therefore, the comparative study of the histogenetic domains that give rise to cortical structures allows us to reconstruct the main features of cortex-evolution, identifying similarities, which potentially may be ancestral features, and differences resulting from independent evolution (Puelles et al., 2000; Abellán et al., 2014; Medina et al., 2017, 2021; Puelles, 2017, 2021; Desfilis et al., 2018).

### Cytoarchitectonic organization of the medial pallium

The cytoarchitectonic analysis of the MP of the species selected for this study shows the cellular arrangement of this area, both in adults and during development, allowing us to visualize the degree of expansion and development that this territory undergoes during the stages of development up to adulthood. This territory, particularly in embryonic and early larval stages in *Xenopus laevis*, shows a very low degree of expansion, being composed of two or three sheets of cells including the ventricular layer (see Figure 1). In *Trachemys scripta*, at stage 17 the expansion of the MP is very limited with no obvious lamimar formations, only a thick ventricular zone (vz) and a very small population of cells in the mantle (see Figure 2). In agreement, in the lizard *Psammodromus algirus* developing pallium, it has been described during the development that the medial cortex (MCx) is the most immature domain, showing a very thick vz and mantle (Desfilis et al., 2018). In *Xenopus laevis*, later on, at larval prometamorphic stages, this medial domain begins to undergo a significant degree of enlargement [(Moreno and González, 2017); present results]. At these stages, its anatomical appearance resembles that found in adults, showing a columnar cellular organization, orthogonally situated in relation to the radial glia fibers (present results). Thus, in *X. laevis* it is from larval stages when the expansion of this region happens, temporally corresponding with the second wave of neurogenesis described in the pallium (Moreno and González, 2017). In *Trachemys scripta*, at stage 26 the arrangement is close to that observed in adults, clearly showing layers (present results), that in combination with the radial glia analysis, define that the main cellular layer is located orthogonal to the radial glia fibers, and therefore parallel to the ventricular surface (present results). This timing is very interesting from a functional point of view. We lack data on the behavior of developing turtles, but in *Xenopus laevis*, in embryos between stages 32 and 37/38, in which, based on our data, the MP expansion has not yet begun [(Moreno and González, 2017); present results], simple mechanisms derived from contact reflexes (non-vestibular), mediate the orientation of scape swimming response (Roberts et al., 2000), such this behavioral response begins to be seen at this point in development (Zahn et al., 2022). Therefore, the functional/behavioral capabilities of this model, attributed to the MP (see above), could (i) start to operate at later stages of development, or (ii) it could be the additional territories, already specified at these stages, functionally involved in this task, or (iii) even with the MP at a low degree of development, it could already be involved in these functions. Therefore, more combined analyses during development are needed to identify exactly the functions in which this area is involved.

As explained before, in *Xenopus laevis* there is no pallial laminar organization or nuclei [although they do exist in other areas of the brain; see Nieuwenhuys et al. (1998)]. The cells show a columnar arrangement that perpendicularly follows the extension of the radial glia (present results). This model of no laminar organization is also the one traditionally described in birds, although in this case, it has been postulated that their development does follow a laminar cytoarchitectonic pattern, even postulated in adults [(Redies et al., 2001; Suárez et al., 2006); see discussion in Medina et al. (2017)]. Moreover, and it has been described that the connectivity of this region respects the layering pattern (Atoji and Wild, 2004). In anurans, a layered organization, or any kind of cellular arrangement has not been clearly described, although there are some data. In the classic review of the pallium of anuran amphibians by Neary, indicated that the segregation of the neurons projecting to the olfactory bulb suggests the presence of an external MP layer, and connectivity studies further indicate that there are adjacent inner and outer cellular layers, almost impossible to delineate (Neary, 1990). Moreover, this would be supported by the identification of different cell types particularly located (Clairambault and Derer, 1968; Northcutt and Ronan, 1992; Westhoff and Roth, 2002). Regarding it, based on the detailed analysis of the studies of Roth and cols (Roth and Westhoff, 1999; Westhoff and Roth, 2002; Laberge and Roth, 2007; Roth et al., 2007; Laberge et al., 2008), in which they make very precise camera lucida reconstructions of the projection neurons, the location of the projection cells can be clearly visualized, close to the periventricular zone, sending their axonal projections and dendrites externally toward the most distant zone of the mantle. The pattern of projections described toward this zone supports this organization, since projections are mostly localized in this outermost zone where the cellular extensions of the projecting neurons arrive (Westhoff and Roth, 2002; Laberge and Roth, 2007; Laberge et al., 2008). In particular, in the analyses of the anatomy of the anterior thalamo-telencephalic pathway in the frog *Bombina orientalis*, it is observed how the projection occupies the outermost part of the MP region [see Figures 8A–E from Laberge and Roth (2007)]. Finally, the recently described pattern of interneuron distribution fits well with this pattern of zonal organization (Jiménez et al., 2020).

### Specification and development of the medial pallium

In mammals, there have been described two secondary organizers acting in the development of the pallium, medially the cortical hem (CH) and laterally, at pallial-subpallial boundary, the antihem (Subramanian et al., 2009; López-Mengual et al., 2022). It has been demonstrated that the CH in mammals induces the formation of the hippocampus in the adjacent neuroepithelium, thus, defining its anatomical location. In particular, the loss of CH or one of its main morphogens, Wnt3a, results in the loss of the hippocampus (Lee et al., 2000). Similarly, its hippocampal-inducing capacity has been demonstrated by ectopic donations analysis (Mangale et al., 2008). Numerous factors determine the function of this organizer, but in concrete Lhx2 it is a suppressor of CH, since in its absence it expands (Bulchand et al., 2001; Mangale et al., 2008). Furthermore, in mammals together with Lhx2, Pax6 expression is essential in the anatomical localization of CH (Godbole et al., 2017) and thus of the hippocampus. In *Xenopus laevis*, the presence of a CH has recently been defined based on the expression of different conserved genes, including Wnt3a (Jiménez and Moreno, 2021), as well as the Lhx2 and Pax6 pallial expressions [(Bachy et al., 2001, 2002b; Moreno et al., 2004, 2008); present results]. Moreover, in mammals it has been demonstrated the involvement of the Wnt signaling pathway in the Prox1 + granule cells regulation (Liu et al., 2000; Pleasure et al., 2000; Gao et al., 2009; Kuwabara et al., 2009; Karalay et al., 2011). Therefore, the Wnt3a expression in the *Xenopus laevis* CH (Jiménez and Moreno, 2021), together with the Prox1 expression in the MP (present results), suggest that the molecular developing specification process in both models could be comparable. In reptiles, a basic and small CH has been described [(Cabrera-Socorro et al., 2007; Nomura et al., 2013), as well as the Lhx2 and Pax6 expressions in MP (Moreno et al., 2010; Desfilis et al., 2018; Tosches et al., 2018); present results]. Therefore, also it would be expected that the participation of Lhx2 in the specification of this territory, as well as in the blocking of CH, may be conserved in evolution, as are the expressions of the main markers [(Jiménez and Moreno, 2021); present results].

In terms of the development of the HF components in mammals, particularly the DG constitutes a unique structure with a complex neurogenesis that continues throughout life (Radic et al., 2017). The present results, in agreement with previous analysis of the pallial progenitors in *Xenopus laevis*, demonstrate that at early developmental stages the ventricular expressions of Lhx2, Pax6, BLBP/GFAP and Sox2 define pallial primary precursor cells in the MP, identified as radial glial cells [(Moreno and González, 2017); present results]. Moreover, the mitotic abventricular Sox2/DCX cells described [DCX is expressed in both progenitor cells and immature neurons; (Kempermann et al., 2004)] correspond, among other populations, to intermediate progenitors (Moreno and González, 2017). Similarly, later in development, at prometamorphic stages Lhx2/Pax6 expressing cells could represent new intermediate progenitors that have just abandoned the vz [(Moreno and González, 2017); present results]. From these stages this abventricular expression, in what could be intermediate progenitors or just new postmitotic cells, coincides with the moment of expansion of this territory (present results) and with the second wave of neurogenesis (Moreno and González, 2017). Recent RNAseq analyses in the brain of *Pleurodeles waltl* demonstrate that during development the pallial region follows two differentiation trajectories, one dorsomedial and one ventrolateral (Woych et al., 2022). As shown in developing *Xenopus laevis* pallium based on early expression of LIM-hd family genes [see Bachy et al. (2001)]. This dorsomedial stream is characterized by the expression of Zbtb20, Lhx9, Fez2, ER81 and Prox1 (Woych et al., 2022), all of them described in the brain of *Xenopus laevis* [(Bachy et al., 2001, 2002b; Moreno et al., 2004); present results; discussed below]. The turtles are the only reptiles in which have been defined intermediate progenitors, but mainly in the ventral pallial domain (Clinton et al., 2015; Martínez-Cerdeño et al., 2016). In the present analysis, in particular, Lhx2 and Pax6 are expressed in the vz of the MP, in agreement with what has been previously described [see Figures 3, 4 in Moreno et al. (2010) and Tosches et al. (2018)]. Moreover, in concordance to that, during the lizard development, notably in *Psammodromus algirus*, the observed Lhx2 expression pattern closely resembles that observed in *Trachemys scripta* [see Figure 4K of Desfilis et al. (2018)], described as moderate in the MCx cortical plate and dorsomedial cortex (DMCx) and light in the dorsal cortex (DC), however, there are no data on its expression in adult (Desfilis et al., 2018).

### The medial pallium derivatives: Comparison with the main components of the hippocampal formation

**FIGURE 13:**
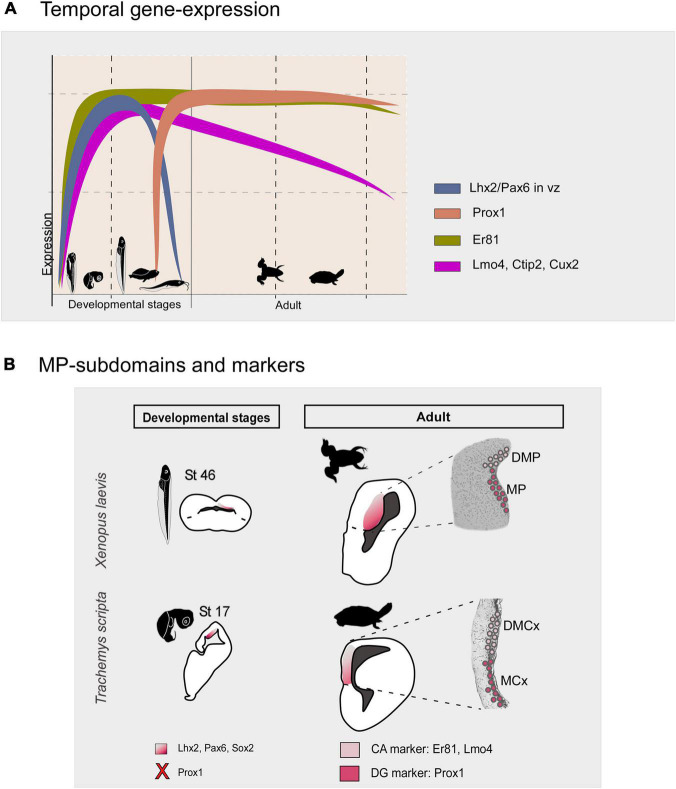
**(A)** Summary diagram of the temporal gene expression of the markers analyzed in this study in the medial pallium of *Xenopus laevis* and *Trachemys scripta elegans*. **(B)** Schematic representations in development and adult showing that there are two subdivisions in both models, the MP/MCx: Prox1 + and the DMP/DMCx: Er81/Lmo4 +, related to mammalian DG and CA respectively. The X in the diagram indicates the lack of Prox1 expression at these developmental stages. The color code is indicated in each figure. Silhouette images of adult animals were obtained from *phylopic.org*. See the abbreviation list.

In the present analysis, we identified in both models differential expressions of some conserved transcription factors, both in development and in the adult, that allow us to propose a basic bipartite organization of this pallial domain, and postulate similarity relationships with their counterparts in other vertebrates (see Figures 13A,B). Specifically, from middle developmental stages and adults, the combination of Prox1, Er81 and Lmo4 allow the identification of DG-like and CA fields. We found cells expressing Prox1 + in a specific subdomain of the Lhx2 territory, even observing double-labeled cells among abventricular postmitotic cells (present results). In mammals, Prox1 is frequently used as a lineage marker of DG granule neurons. It plays an essential role in the neural progenitors and granule cells DG proliferation (Rubin and Kessaris, 2013) and it is expressed in postmitotic granular cells (Liu et al., 2000; Pleasure et al., 2000; Lavado et al., 2010; Iwano et al., 2012; Hodge et al., 2013; Atoji and Sarkar, 2018). Thus, given that in mammals Prox1 is essential in the development of the DG (see above) and it is not expressed in the rest of the HF (except for scattered cells), we conclude that the MP in *Xenopus laevis* and the MCx in *Trachemys scripta* likely constitute, in both cases respectively, the DG-like domain (see Figure 13). Regarding the expression of Er81 and Lmo4, in amniotes both transcription factors during late development are enriched in the CA and subiculum fields (Gray et al., 2004; Abellán et al., 2014), in particular, both show higher expression intensity in CA3 [see discussion in Medina et al. (2017)]. In our analysis, in both models Er81 and Lmo4 are absent, or poorly expressed in the ventricular region of the Prox1 + territory, in contrast to the adjacent region, the DMP in *X. laevis* and the DMCx in *T. scripta*. Therefore, the DMP and the DMCx could be related, in both cases respectively, to the CA3-like domain (present results; see Figure 12B). Concerning the additional transcription factors analyzed, described in HF of mammals (Nielsen et al., 2013), in both models we observed consistent and coincident expression patterns (present results). In particular, in mammals, Bcl11b/Ctip2 is postnatally expressed by granular cells of the DG and CA (Simon et al., 2012, 2016). In both models, we observed during development Ctip2 expression in the two proposed domains, but it is in the MP and MCx region, respectively, where we observed this expression in the ventricular zone and where it is maintained in the adult (present results). This variable expression during adult vs development may be related to the involvement of Ctip2 during early development in progenitor cell proliferation, specific differentiation of granule neurons and functional integration into the hippocampal circuitry (Simon et al., 2012, 2016). In particular, in the markers we analyzed in both models we observed a predominant expression in the DG-like regions, the MP and MCx, at late developmental stages (present results). In *Xenopus laevis*, this is confirmed by the observation of double-labeled Satb1/2-Prox1, Cux2-Satb1/2 cells (present results). In *Trachemys scripta*, the Satb1/2 cell population is no longer expressed in adult individuals (present results). Furthermore, in agreement with our results in *Xenopus laevis* (present results), the presence of Mef2c expressing cells in the mammalian hippocampus (Hain et al., 2022) and in the medial cortex of reptiles (Tosches et al., 2018; Hain et al., 2022), as well as EGFR cells in the mammalian hippocampus, involved in the maintenance of progenitors, have been described (Aguirre et al., 2010; Galvez-Contreras et al., 2013).

Recent RNAseq studies have constituted a new and very important source of information in the understanding of the pallium (Faltine-Gonzalez and Kebschull, 2022). Specifically, in dorsomedial cortex of turtles, the combined analysis of mammalian hippocampal markers such as ZBTB20 + together with Prox1 and Mef2c identify the medial cortex; while Er81, Meis2 and Lmo4 identify the dorsomedial cortex, showing overlap with their mammalian counterparts, DG and CA respectively (Tosches et al., 2018). These studies also suggest that the posterior dorsomedial cortex may be related to the subiculum (Tosches et al., 2018).

Similarly, recent RNAseq analyses in developing *Pleurodeles waltl* demonstrate that the MP expresses hippocampal-specific transcription factors (Woych et al., 2022). The authors propose that the vast majority of *P. waltl* medial and dorsal pallium neurons show similarities to mammalian hippocampal, EC and subiculum neurons (Woych et al., 2022), in both cases, in full agreement with our present results.

### The boundaries of the medial pallium: Implications for understanding the evolution of the dorsal cortex

The classic review on the pallium of anurans indicated that in this field (the larger pallial region, rostrally dorsal to the postolfactory eminence and from medial levels separated from the septal nuclei by the cell-poor zona limitans medialis, and bounded dorsolaterally by the dorsal pallium), there were no obvious changes in cell size or Nissl staining properties (Neary, 1990). However, although histologically not easy to recognize, it was suggested that it could divide in a transitional part, the dorsal part, a large cell part, centrally, and a small cell part [see review in Neary (1990)]. In a comparative study in the frog *Discoglossus pictus* and the salamander *Plethodon jordani* on the connections of the medial and dorsal pallium, the authors propose the subdivision of the MP into a ventral and a dorsal portion, and the DP into medial and lateral domains [see Figure 21 of Westhoff and Roth (2002)]. At this point, it seems clear that in addition to the basic bipartite organization proposed (present results), there are adjacent regions whose origin and evolutive relationships are unknown and should be the subject of analysis. This adjacent territory in *Xenopus laevis*, in addition to showing Lmo4 expression, shows a significant reduction in Er81 (present results). In mammals, it has been described that CA1 expresses Lmo4, in contrast to Er81, which shows moderate expression [see Medina et al. (2017)]. Therefore, it is possible that at least part of the *Xenopus laevis* dorsal pallium in fact belongs to the medial domain, since, in amphibians there is no convincing argument for a clear distinction between medial and dorsal pallium, but traditionally it has been localized as a narrow dorsal band between the medial and lateral regions (Northcutt, 1974; Kicliter and Ebbesson, 1976). In amniotes the dorsal pallium express Lhx2 in the vz and the mantle (Rétaux et al., 1999; Abellán et al., 2014; Desfilis et al., 2018), like in *Xenopus laevis*, that during development, the expression of Lhx2 in the ventricular region is widespread in both pallial territories, as for the expression of other pallial transcription factors such as Tbr1 and Emx1 (Bachy et al., 2001, 2002a; Brox et al., 2003, 2004; Moreno and González, 2017). Therefore, at the moment, there is no molecular evidence to identify both territories with certainty, except for the noticeable decrease in Er81 expression in the mantle of what we identified as DP [(Bachy et al., 2001; Brox et al., 2004); present results]. Hodologically, the dorsal pallium projects exclusively to the ipsilateral medial pallium and the dorsal border of the lateral pallium (Westhoff and Roth, 2002), which despite not allowing a clear histological identification/delimitation, constitutes a distinction, which allows hypothesizing that this pallial region constitutes the only intrapallial connecting region. It might allow to suggest that this region could be compared to CA loops described in mammals [see discussion in Medina et al. (2017)], supporting its involvement in HF. Moreover, this closely resembles the situation described in lizards (Desfilis et al., 2018) and turtles [present results; (Tosches et al., 2018)]. In this line, as indicated above, recent RNAseq analyses in *Pleurodeles waltl* showed that the dorsal pallium is devoid of cellular and molecular characteristics of the mammalian neocortex, and the authors (despite noting that the results of the pallium were more ambiguous than those of other regions), propose that it shares similarities, including its hodology, with EC and subiculum of mammals (Woych et al., 2022). This implies the lack of a proper dorsal pallium, as a histogenetic domain in *P. waltl*, which may be due to secondary loss/simplification (Roth et al., 1993, 1997), or that this feature appears at the base of amniotes and is absent in anamniotes.

In this line, a recent proposal in *Passodrommus algius* includes as part of the MP derivative the MCx, DMCx and most dorsal cortex [DC1 + DC2; see Desfilis et al. (2018)]. These regions would be compared, respectively, to the DG, the CA3 and the EC of mammals and to the ventral hippocampus or v-field area and the parahippocampal area of birds [(Abellán et al., 2014); and see Discussion in Medina et al. (2017)], and with the CA and part of the subiculum of turtles [see discussion in Tosches et al. (2018)]. Moreover, the region of lizard identified as DC2 has particularly been related to the EC (Desfilis et al., 2018). Like in birds, based on the expressions of Lef1, Lhx2 and Lhx9 during development, and its olfactory inputs (Abellán et al., 2014; Medina et al., 2017). Thus, in *X. laevis*, this domain, adjacent to the present DMP, could be part of the MP, since among other features express Lhx2 and receives olfactory projections (Northcutt and Ronan, 1992), as the EC.

However, there are other possibilities, and this area may be implicated as an intratelencephalic integrative multisensory center, which functionally would be more like a frontotemporal cortex. In this line, in *Bombina orientalis*, thalamic inputs to the medial and dorsal pallium have been demonstrated [see Westhoff and Roth (2002), Roth et al. (2003), and Laberge et al. (2008)] and some of these terminals reach in the MP the exact position of the DP projections, thus constituting an integrative loop [see discussion of Westhoff and Roth (2002) and Roth et al. (2003)]. In this line, Roth et al. (2007) describe the existence of a rostral pallium, defined by the presence of cells with rather “*chaotic*” dendritic arbors and by receiving most of the inputs from the anterior dorsal thalamus. In addition, further electrophysiological and *c-Fos* studies suggest that the rostral pallium of anurans is a multimodal sensory integration center, possibly with both sensorial and cognitive functions [see discussion in Roth et al. (2007)].

Summarizing all this information, what seems clear is that it constitutes a distinct region hodologically (Westhoff and Roth, 2002), neurochemically [see discussion in Northcutt (1974)] and genoarchitectonically [present results; (Woych et al., 2022)]. Based on our results, this bordering region could be part of the MP derivatives, but further analysis of this region is necessary, given its degree of complexity and the important evolutionary consequences of establishing similarities in this domain.

## Conclusions

As a general pattern, normally in the early stages of development the brain subdivisions show a more similar transcription factor expression pattern, whereas variations occur later in development. In the case of our results in *Xenopus laevis* and *Trachemys scripta elegans* what we observe during development is that the major progenitor cells, the radial glial cells, the main transcription factors involved in region specification, such as Lhx2 and Pax6, and probably the secondary organizer, the cortical hem, are conserved (see discussion; Table 3). As development progresses, in late stages, when in both cases the brain morphology resembles that found in the adult, can be observed a Prox1 + /ER81- DG-like region, the MP and the MCx respectively, and a CA3-like Er81/Lmo4 + domain, the DMP and the DMCx respectively (see Figure 13 and Table 3). In agreement with the main major cell types and gene specification networks described by RNAseq analysis in *Trachemys scripta* and *Pleurodeles waltl* (Tosches et al., 2018; Woych et al., 2022). Therefore, the previous proposal about the DG as a novel mammalian acquisition (Striedter, 2016), does not fit with our results, which are in agreement with what has been described in chicken and other reptiles (Abellán et al., 2014; Desfilis et al., 2018; Tosches et al., 2018). In this, based on mammalian Prox1 inactivation experiments, it appears that DG and CA3 may evolve from a common domain that is differentially specified later (Iwano et al., 2012).

**TABLE 3:** Summary table showing the main expression patterns found for the genes analyzed in the present study, in the middle stages of development and in the adult, in the ventricular zone and out of the ventricle.

	Mid developmental stages		Adult	
	*vz*	*mz*	*vz*	*mz*

**MP** 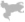	Lhx2	Lhx2	–	Lhx2
	Prox1	Prox1	–	Prox1
	–	Er81	–	Er81
	–	–	–	–
	Ctip2	Ctip2	Ctip2	Ctip2
	*Cux2?*	*Cux2?*	Cux2	Cux2

**MCx** 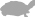	Lhx2	Lhx2	*Lhx2?*	*Lhx2?*
	Prox1	Prox1	Prox1	Prox1
	*Er81?*	*Er81?*	–	Er81
	–	–	–	–
	–	Lmo4	–	Lmo4
	Ctip2	Ctip2	Ctip2	Ctip2
	Cux2	Cux2	Cux2	Cux2

**DMP** 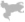	Lhx2	–	–	–
	–	–	–	–
	–	Er81	Er81	Er81
	–	Lmo4	–	Lmo4
	–	Ctip2	–	Ctip2
	*Cux2?*	*Cux2?*	–	–

**DMCx** 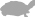	Lhx2	–	*Lhx2?*	*Lhx2?*
	–	Prox1	–	–
	*Er81?*	*Er81?*	–	Er81
	Lmo4	Lmo4	Lmo4	Lmo4
	–	Ctip2	–	Ctip2
	–	Cux2	–	Cux2

**DP** 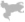	Lhx2	–	–	–
	–	–	–	–
	–	–	–	–
	–	Lmo4	–	Lmo4
	Ctip2	Ctip2	Ctip2	Ctip2
	*Cux2?*	*Cux2?*	–	Cux2

**DCx** 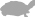	Lhx2	–	*Lhx2?*	*Lhx2?*
	–	–	–	–
	*Er81?*	*Er81?*	–	Er81
	Lmo4	Lmo4	Lmo4	Lmo4
	–	Ctip2	–	Ctip2
	–	Cux2	–	Cux2

Those markers shown in gray present low expression and/or in scattered cells. Those markers in italics with a question mark have not been analyzed at the indicated stage.

Therefore, this would suggest that the basic genetic networks controlling the early specification of this territory, and of some of its subdomains, are present from the anamniotes, although anatomical divergences are evident and there are other notable differences during the process of cortical development in early stages, such as the presence or absence of intermediate progenitors (Moreno and González, 2017; Nomura et al., 2018; Docampo-Seara et al., 2020).

In this context of analyzing specific characteristics from an evolutionary point of view, according to evolutionary theory, observable similarities may be consistent with homology (understood as a hypothesis corroborated by cladistic analysis), but it is not necessarily synonymous. The ultimate argument for homology, in addition to the basic correspondence of bauplan and development (which are necessary, but not sufficient) is phylogenetic continuity [see Northcutt (1990)]. And in this context, in the absence of a specific cladistic analysis of the details of the medial pallium, the present study supports the hypothesis of homology of the medial pallium in tetrapods, filling in the gaps in this story in an expected way, since to date no vertebrate group shows in a cladistic analysis the lack of a medial pallium. However, the question remains open as to when the subdivision between the two domains originated in evolution, since we now know that it is present at least since amphibians (present results). After all, the analysis and identification of the different subdivisions of the HF and even more the functions in which each of them is involved in mammals, and thus in all vertebrates, is still an open field [see for example Bienkowski et al. (2018)]. Thus, as a general reflection, it is worth noting the large number of questions that arise and remain unanswered from these comparative analyses, as we still do not know whether the cell types that appear in the conserved histogenetic domains have the same functions in the cortical circuits of the different species, for example.

## Data availability statement

The original contributions presented in this study are included in the article/supplementary material, further inquiries can be directed to the corresponding author.

## Ethics statement

The animal study was reviewed and approved by the Complutense Animal Ethics Committee: ES-28079-0000086.

## Author contributions

NM devised the study and wrote the manuscript. SJ performed the experiments. NM and SJ analyzed the results, prepared the figures, and corrected and edited the manuscript. Both authors had full access to all the data in the study and take responsibility for the integrity of the data and the accuracy of the data analysis and approved the manuscript.

## Abbreviations

BG: basal ganglia
BST: bed nucleus of the stria terminalis
CeA: central amygdala
CH: cortical hem
ChP: choroid plexus
cpal: pallial commissure
DCx: dorsal cortex
DMCx: dorso-medial cortex
DMP: dorso-medial pallium
DP: dorsal pallium
DVR: dorsal ventricular ridge
LCx: lateral cortex
LP: lateral pallium
MCx: medial cortex
MeA: medial amygdala
MP: medial pallium
oc: optic chiasm
Pa: pallium
pa: pallidum
POA: preoptic area
PT: pallial thickening
S: septum
Sd: dorsal septum
SPa: subpallium
Str: striatum
SPV: supraoptoparaventricular area
Th: thalamus
v: ventricle
vz: ventricular zone
VP: ventral pallium.
